# The Induction of Bone Formation: The Translation Enigma

**DOI:** 10.3389/fbioe.2018.00074

**Published:** 2018-06-07

**Authors:** Roland M. Klar

**Affiliations:** Laboratory of Biomechanics and Experimental Orthopaedics, Department of Orthopaedic Surgery, Physical Medicine and Rehabilitation, University Hospital of Munich (LMU), Munich, Germany

**Keywords:** bone induction, biomaterials, gene and protein expression, compositional requirements, animal to human translation, bone grafting, enigma

## Abstract

A paradigmatic shift in the way of thinking is what bone tissue engineering science requires to decrypt the translation conundrum from animal models into human. The deductive work of Urist (1965), who discerned the principle of bone induction from the pioneering works of Senn, Huggins, Lacroix, Levander, and other bone regenerative scientists, provided the basis that has assisted future bone tissue regenerative scientists to extend the bone tissue engineering field and its potential uses for bone regenerative medicine in humans. However, major challenges remain that are preventing the formation of bone by induction clinically. Growing experimental evidence is indicating that bone inductive studies are non-translatable from animal models into a clinical environment. This is preventing bone tissue engineering from reaching the next phase in development. Countless studies are trying to discern how the formation of bone by induction functions mechanistically, so as to try and solve this enigmatic problem. However, are the correct questions being asked? Why do bone inductive animal studies not translate into humans? Why do bone induction principles not yield the same extent of bone formation as an autogenous bone graft? What are bone tissue engineering scientists missing? By critically re-assessing the past and present discoveries of the bone induction field, this review article attempts to re-discover the field of bone formation by induction, identifying some key features that may have been missed. These include a detailed library of all proteins in bones and their arrangement in the 3D superstructure of the bone together with some other important criteria not considered by tissue engineering scientists. The review therefore not only re-iterates possible avenues of research that need to be re-explored but also seeks to guide present and future scientists in how they assess their own research in light of experimental design and results. By addressing these issues bone formation by induction without autografts might finally become clinically viable.

## Introduction

“*An individual unwilling to change is doomed*” (Albert Einstein). Indeed much of science is based upon the principle of change and adaptation with theories and paradigms prone to change and alterations until complete perfection, a scientific unlikelihood, is attained. It is this basis that has ensured that science and society as a whole has reached the technological standard that is so common in modern day life. However, before change is accepted it takes decades if not centuries, as adapting current philosophies remains difficult. Yet, without the necessary stimulus current processes cannot attain the necessary quality to become beneficial or useful and indeed the prospect of inducing bone formation finally in humans is no different.

The paradigm of bone induction has come a long way. But still this form of tissue regeneration is in its infancy. It may seem like the field has progressed far but in reality prosthetics is more advanced than any present form of regenerative tissue science. After more than a century clinical bone repair and regenerative procedures still rely on the autogenous bone graft, discovered by Havers (1692), Ollier (1867), and Senn (1889). Whilst some progress has been made in our understanding of the molecular mechanisms and how bone formation can be induced (Huggins, 1931; Levander, 1938; Lacroix, 1945; Urist, 1965; Ripamonti, 1990, 1991; Wang et al., 1990; Reddi, 1994, 2000; Ripamonti et al., 1997, 2000, 2008; Klar et al., 2014) including the requirements to induce bone formation via the soluble and insoluble combination paradigm (Sampath and Reddi, 1981), it still remains a vision to successfully induce, form and attain the extent of new bone formation similar to that of an autograft. The question one must therefore ask is what has been missed?

As science bases itself on constantly adapting its principles, it may be time to tackle the problem of the non-bone-inductive clinical scenario in a different manner. To do this the literature over the last century was re-assessed, looking at key discoveries and present research to determine criteria that have either been ignored or still need to be answered that so far have not been considered yet. Only the most important material was selected and drawn upon to design possible scenarios with relevant answers that the author of this article strongly believes are in his opinion the solutions that could finally answer the translation enigma from animal to human and bone induction in medicine as a whole. But much like Einstein gravitational theory, proven by Eddington (1920), these hypotheses/theories inevitably relies on the support of the scientific community to accept and prove their validity. If not, bone regenerative science in a clinical setting will remain but an intention instead of becoming a viable clinically applicable and acceptable process in medicine.

## Osteogenesis and remodeling

Bone, a hard and rigid constituent of the skeleton of vertebrates is composed primarily of calcium salts and connective tissue. Osteogenesis, or bone formation, is the process by which new bone is formed. There are two major modes of bone formation. The conversion of mesenchymal tissue, during embryogenesis, into bone is termed intramembranous ossification (Gilbert, 2000). This process occurs primarily in the bones of the skull and some parts of the face. On the other hand endochondral ossification, a process by which most other skeletal bones are formed, involves first the formation of a cartilage intermediate that is then converted into bone by bone forming cells—osteoblasts (Gilbert, 2000).

Once bone has fully developed the only mode to generate new bone is via the remodeling or regeneration cycle. This fascinating and continuous process becomes active once neonatal life commences and remains metabolically operational until the end of the vertebrate's existence. The bone remodeling cycle was first described by Eriksen (1986) and involves a series of specialized cellular events. The mesenchymal osteoblastic lineage and the hematopoietic osteoclastic lineage are the two major constituents necessary for proper bone regeneration (Raisz, 1999). As such, resorption and formation exist within a balanced homeostatic equilibrium, in which timeworn bone is continuously replaced by new osteogenic material, helping to not just maintain the integrity and health of bone but also permit bone to adjust to mechanical loads and strains (Frost, 1990). Though there is still much debate on what exactly initiates the remodeling cycle, calcium ion dissociation from the bone matrix (Boyle et al., 2003; Caudarella et al., 2011; Hwang and Putney, 2011; Boyce et al., 2012; Klar et al., 2013) and bone initiatory growth factor release via osteocytes (Hadjidakis and Androulakis, 2006) within the bone have been suggested to be key events linked to the bone regeneration process. However, how they interact and the chronological order that is followed as the signals interact with each other still remains unclear.

Osteoclasts and osteoblasts closely cooperate with each other in the remodeling process in what is called the basic multicellular unit (BMU) (Hadjidakis and Androulakis, 2006). The BMUs are organized differently throughout cortical and trabecular bone. Within the cortical bone the BMU cuts a cylindrical canal of approximately 2,000 μm in length and 150–200 μm in width within the bone, at an estimated rate of 20 μm per day. This means that during 24 h, ~10 osteoclasts can excavate a circular tunnel (Petrýl et al., 1996), which is filled in with new bone via several thousands of osteoblasts (Parfitt, 1994). Thus per annum about 2–5% of cortical bone is remodeled. In contrast to this, trabecular bone is more actively remodeled than cortical bone. For trabecular bone the surface to volume percentage is much greater (Hadjidakis and Androulakis, 2006). This means that osteoclasts are capable of moving across the trabecular bone surface at speeds of nearly 25 μm per day, excavating concavity like trenches at an approximate depth of 55 μm (Hadjidakis and Androulakis, 2006) allowing bone remodeling to occur efficiently and effectively within a relative small amount of time.

The remodeling cycle consists of three main consecutive phases: Resorption, reversal and formation. In the first stage of remodeling/bone regeneration, partially differentiated pre-osteoclasts migrate to the surface of the bone directed there either by calcium ion release (Boyle et al., 2003; Caudarella et al., 2011; Hwang and Putney, 2011; Boyce et al., 2012; Klar et al., 2014) or growth factor stimuli released by dormant osteocytes (Hadjidakis and Androulakis, 2006). Pre-osteoclasts complete their transition into multinucleated giant cells and attach to the bone surface that is to be resorbed. An acidic pH is created in the zone of resorption by the osteoclasts which secrete hydrogen ions together with lysosomal enzymes, primarily cathepsin K. This acidic environment ensures that all of the components that make up the bone matrix are dissolved (Väänänen et al., 2000). The resorption process by osteoclasts produces irregular scalloped cavities on the bone surface, which are referred to as Howship lacunae, or cylindrical Haversian canals in the cortical bone (Gilbert, 2000; Hadjidakis and Androulakis, 2006). After the completion of osteoclastic resorption, the reversal phase is entered. Osteoclasts enter apoptosis and mononuclear cells appear on the bone surface. These cells polish the surface of the resorbed area by stimulating further degradation of the collagen matrix within bone. Various proteoglycans are deposited onto the surface to form a cement line whilst at the same time releasing and depositing growth factors such as BMPs and transforming growth factor-β (TGF-β) isoforms (Parfitt, 1994; Gilbert, 2000; Ripamonti et al., 2010), which together help initiate the differentiation and proliferation of osteoblasts that deposit new osteoid at the site of the osteoclastic cut concavities (Chen et al., 2012). In the final formation phase osteoblasts, which have differentiated from their mesenchymal precursors, deposit osteoid that is then transformed into mineralised bone until the concavity has been repaired. Once this stage has been completed the surface of the site of new bone within pre-existing bone is covered with a flattened lining of cells and a prolonged quiescent phase begins until the regeneration cycle is re-initiated (Parfitt, 1994; Gilbert, 2000).

This unique feature of bone, capable of revitalizing itself, has fascinated scientists for centuries. It is this one aspect, which has led to the development of bone regenerative medicine and the exploration to decipher this mechanism, such that damaged tissue can be repaired or completely regenerated.

## Bone autografting

As early as 2630–2611BC the father of medicine, Imhotep, described in the Edwin Smith Papyrus, that bone was able to repair itself when damaged (Shehata, 2004). Later Hippocrates (460–370BC) would add to this initial finding in discovering that bone was capable of regenerating itself with no apparent scaring (Lanza and Vegetti, 1971). These two fundamental discoveries provided the foundations for the emergence of two important scientific fields, i.e., that of tissue regeneration and subsequently the emergence of biomaterial science.

The concept of using bone grafting arose from Havers (1692), who deciphered the inner structures of bone, especially the vascularization of the periosteum. Havers (1692) research permitted other medical specialist to develop techniques in which vertebrate bone, exogenous or endogenous, can be utilized to repair a defect within a damaged area of the skeleton.

Pioneering bone grafts where first performed by Ollier (1867). Ollier conducted a great deal of research on how bone grows and was the first to suggest that a possible way to regenerate bone was to stimulate a patients own cartilage to ossify (Ollier, 1867). Further pioneers to use bone grafting techniques, especially within humans would be Sir William Macewen (1848–1924) and Sir Robert Jones (1855–1924) (Khatiwada, 2012).

Bone grafting is defined as the transplantation of bone from one site to another (Kaveh et al., 2010). Various bone grafting procedures exits but the golden standard of bone grafting and bone regeneration is the autogenous bone graft technique. The autogenous bone graft involves the harvesting of bone, from an individual, and transplanting it within a skeletal site of the same individual (Fox, 1984; Bauer and Muschler, 2000; Zamprogno, 2004). It is deemed the golden standard of bone grafting as it possess key characteristics that whilst known are not considered in their totality when it comes to replicating this during bone induction. Autogenous bone grafts are known to be successful because they contain the highest number of viable osteoprogenitor cells, are comprised of non-collagenous matrix proteins and growth factors that are osteoinductive. Furthermore, the autogenous bone carries with it bone mineral and collagen which provide the foundation for the means for proper osteoconduction (Ladd and Pliam, 1999; Keating and McQueen, 2001; Betz, 2002; Linovitz and Peppers, 2002). Once the autogenous bone graft is transplanted it is incorporated quickly as it is completely biocompatible, within the grafting site and causes a minimal immunological response, which leads to faster healing and recovery (Samartzis et al., 2005). However, there are limitations regarding the autogenous bone grafting.

Depending on where the initial bone is harvested from, in the autogenous graft procedure, various complications are known to develop which can compromise the health of the patient. Minor complications that have been reported are persistent donor-site pain (Summers and Eisenstein, 1989; Goulet et al., 1997; Schnee et al., 1997; Ebraheim et al., 2001), superficial injury of nerves (Smith et al., 1984), the formation of hematomas or seromas (Arrington et al., 1996; Westrich et al., 2001) and infection (Banwart et al., 1995; Arrington et al., 1996; Westrich et al., 2001). Major complications that can arise when using autograft protocols are the formation of deep hematomas, which lead to deep infections (Banwart et al., 1995; Arrington et al., 1996; Goulet et al., 1997; Sasso et al., 1998) and necrosis of harvest site when a large graft is utilized for transplantation (Barth, 1893).

These issues arising from autografting and other bone graft procedures forced medicine with the help of science to try and find alternatives to these techniques and were thus the cornerstones that lead to the field of tissue induction and bone engineering.

## The history of bone induction: discoveries and developments

Induction can be defined as an act that sets in motion some course of events. In terms of osteogenesis the “induction” process is defined as setting into motion the events of bone formation within tissue sites not associated with the bone formation event, i.e., muscle tissue not in the vicinity of skeletal bone or organs.

Bone tissue engineering and regeneration has a rich history (Urist, 1965; Reddi, 2000; Ripamonti, 2006). However, foundations are critical as all developed theories, principles, experiments and paradigms bear their origin from some foundation. An incomplete foundation is most often the cause why applications do not translate (Klar, 2011). As such only the most critical advances are described here as all principles in the bone induction are derived from them but they are the key that has shaped the field and predestined all other research, to follow in the excitement, thereby negating critical systematic steps crucial for science to generate answers, critical for medicine to develop treatments from.

The earliest historical innovative record came from the principle discovery of Senn (1889) nearly two centuries ago. This perhaps is the most prominent discovery from which all future bone induction research emanates from. By utilizing decalcified antiseptic bone Senn (1889) performed a series of implantations into skull defects of canines. Subsequently, to his observations that these devices could stimulate new bone formation, later to be phrased as induction (Levander, 1945), he also serendipitously discovered that an embryonic-like tissue often surrounded the implanted decalcified bone matrix. This unique discovery by Senn (1889) indirectly already suggested that the formation of new bone into these devices was the recapitulation of osteogenesis as it occurred during embryonic development (Levander, 1938).

Though one can dispute that Senn's (1889) work was not truly inductive as he implanted devices into bony sites, Huggins (1931) uroepithelial bone induction studies showed that bone formation could be “induced” within non-bony or heterotopic extraskeletal sites. Later Levander (Levander, 1938; Levander and Willestaedt, 1946) showed that partially extracted ethanol-treated bone matrices implanted in heterotopic sites of rats could also induced new bone formation. Levander (1945) and later again Friedenstein (1968) suggested that there was some “substance” that possessed bone forming capabilities, by initiating non-mesenchymal tissue to differentiate and form new bone by either the endochondral or membranous processes. Yet Levander's (Levander, 1938; Levander and Willestaedt, 1946) discovery proved critical as he further substantiated Senn's (1889) theory that the induction of new bone formation in postnatal life was as a result of the recapitulation of embryonic osteogenesis (Levander, 1938). Subsequently, Lacroix (1945) would aptly name Levander (1938) “substance” as “osteogenin” which can be defines as a substance or molecule that possesses the capability to induce new bone formation.

Other discoveries of note are those of Moss (1958) who reported on the osteogenic inductor in bone and Trueta (1963) who discerned that angiogenesis was linked to successfully achieving new osteogenesis. However, it would be the deductive and pioneering work of Urist (1965), who discerned from the pervious works of Senn, Huggins, Levander, Lacroix, Moss, and Trueta together with his own visionary work, the foundations and principles of bone induction (Urist, 1965; Urist et al., 1967). However, as critical as the discoveries may have been they are also indications as to why the induction of bone formation still does not sufficiently form adequate bone in humans. As visionary as Urist may have been, why he believed that only a single molecule or family of molecules initiated or induced bone formation remains a mystery.

In a series of heterotopic intramuscular implantations, using allogeneic demineralized bone matrix, with the rodent and lagomorph models including implantations within calvarial orthotopic defect sites, Urist described to process of autoinduction of bone (Urist, 1965). More importantly, he discerned that there was a “bone morphogenetic protein” complex present within the matrix of the bone that initiated the creation of new bone or as he more commonly referred to it bone morphogenesis. The subsequent experiments in other animals models including *Homo sapiens* finally led to the development and description of a new family of protein members specifically referred to as “bone morphogenetic proteins (BMPs)” (Urist et al., 1967; Urist and Strates, 1971), which were later extracted and cloned by Wang et al. (1988) and Wozney et al. (1988).

Subsequently, Friedenstein (1968) presented findings which showed that certain organs could also be forced into a bone inductive lineage. By transplanting into heterotopic sites, creating surgical lesions in the wall or ligating the renal arteries of urinary bladders this organ could be made to ossify. This further extended the idea of the BMPs being present throughout the various animal tissues, performing different functions during development that could be directed to form new bone, provided certain conditions were met. This work would further contribute to another major discovery, which was the development of the “bone induction principle” (Urist et al., 1967).

With the successful extraction of the BMPs from the extracellular matrix (Reddi and Huggins, 1972; Sampath and Reddi, 1981; Reddi, 1994) a further essential step to achieving the successful induction of bone formation was the discovery that only an extracellular matrix with their morphogens reservoir could induce new bone formation (Sampath and Reddi, 1981). The bone induction principle as it would become known, stipulated that an insoluble matrix carrier and soluble molecular signals in the form of morphogens were required in union to achieve the successful induction of new bone *in vivo* (Sampath and Reddi, 1981). Indeed experimentation in which Sampath and Reddi (1981) dissociatively separated the soluble molecular signals from the insoluble collagenous matrix of demineralized bone, by using chaotropic agents specifically guanidinium hydrochloride and/or urea, neither of the two components separately induced new bone formation heterotopically (Sampath and Reddi, 1981). Only when both components were recombined was the inductive potential restored. Though this provided a reproducible experimental way to test various morphogens for their inductive potential, and indicated that bone was in part a reservoir for BMPs, it is curious why it was assumed that it was the presence of BMPs that induced the bone formation when in fact bone is comprised of more than just BMPs (Klar, 2011). Instead of identifying the total chemical composition, structural and signaling proteins including the various elements present in bone, research was committed to isolating, sequence and clone out BMPs, which were further revealed to be a sub-group of a larger supergene family, i.e., the TGF-β supergene family) (Wang et al., 1988; Wozney et al., 1988; Özkaynak et al., 1990; Reddi, 2000; Ripamonti et al., 2004; Ripamonti, 2006) which are considered the best protein group of inducing new bone formation (Kaur et al., 2016).

Since then, the vast majority of experimental research has tried to clinically induce bone formation in man, which though successful in various animal models, has unfortunately not yet come close to replacing the golden standard for bone tissue regeneration, the bone autograft. Perhaps the solution lies at determining how bone induction functions mechanistically at the gene and protein expression level. On the other hand, there may be a gap in information related to the structural and signaling *milieu* of proteins and other elements that make up bone which have yet to be fully elucidated and interpreted.

## Biomaterials

Biomaterials are defined as synthetic or natural materials that are suitable for utilization in constructing artificial organs or prostheses. Biomaterials can be derived either from nature or by synthetic using a variety of chemical approaches. They are often used and/or adapted for a medical application, and thus comprise whole or part of a living structure or biomedical devices which performs, augments, or replaces a natural function. Function may be benign, such as being used for regenerating a bone segment or may be more bioactive with a more interactive functionality such as hydroxyapatite coated hip implants.

Biomaterials are generally formed by self-assembly. Self-assembly is the most common term used to describe the spontaneous aggregation of particles, without the influence of any external force. Large groups of such particles are known to assemble themselves into thermodynamically stable, structurally well-defined arrays. Molecular self-assembly is found primarily in biological systems and provides the basis for a wide variety of complex biological structures. It is these self-assembled biological structures which are most sought in biomaterials and how to replicate their design since naturally derived composites to date are still the most efficient when it comes to stimulating, initiating or inducing regenerative processes. Though more than 190 different biomaterials exist to date, only a few can induce bone formation via spontaneity; some of which are coralline derived devices (Weber et al., 1971; Chiroff et al., 1975; White et al., 1975; Ripamonti, 1990, 1991).

Corals share a somewhat similar structural, physical and chemical characteristic as that of bone. This trait was first observed by Chiroff et al. (1975). Members of the Porites and Goniopora species both belonging to the Poratidae family of corals, are the best suited at designing coralline calcium phosphate/hydroxyapatite bone substitutes (Chiroff et al., 1975). These corals are effective in promoting bone development within their spaces/pores as the pore sizes, between 200 and 500 μm in diameter, are effective in directing cellular migration and subsequent differentiation of cells associated with the bone formation process. But before corals are able to do so they have to undergo hydrothermal exchange (Roy and Linnehan, 1974) and morphological alterations by replamineformation (White et al., 1975). In the hydrothermal exchange process, coral exoskeletons and phosphate are added in equal quantities to water (Roy and Linnehan, 1974). Under extreme heat and pressure the carbonate ions within the exoskeleton of the coral (calcium carbonate—CaCO_3_) are substituted by phosphate, producing hydroxyapatite (calcium phosphate—CaPO_4_). Varying the amount of phosphate or time taken for the exchange to occur can yield varying conversion, i.e., partial conversion of calcium carbonate to hydroxyapatite, to complete conversion of calcium carbonate to hydroxyapatite. This greatly affects bone formation within the coral derived biomimetic device. Once the hydrothermal exchange has been completed the coral is replamineformed (White et al., 1975), whereby the surface morphology is altered such that the device takes on the shape and structure of bone which can vary anything from mandibular, cranial or long bones, hence mimicking skeletal structures.

Though there are a various substitutes that have been tested as alternatives to bone grafts, none have been more successful at inducing bone formation than coral-derived macroporous devices (Ripamonti, 1990, 1991). Corals have been shown to be of clinical significance. Not only do coral derived devices act similarly to bone grafts used in filling up bone voids resulting from articular surface depressions in tibial plateau fractures (Bucholz et al., 1989) but they are also one of the only biomaterials able to spontaneously induce bone formation without the application of exogenous growth factors (Ripamonti, 1990, 1991; Ripamonti et al., 2010; Klar et al., 2013) and next to the 190+ different biomaterials currently available, with considerable more being added yearly, corals remain to date, in this authors opinion, the best and only viable source of possible bone inductive biomaterial, as it is both osteoconductive and inductive, requires no morphogens to induce bone formation at the site of implantation, irrespective of the defect size, and IF the correct morphogen could be found then the solution to healing bone defects comes that much closer to reality. However, history has shown that even with such good qualities and capabilities something is being missed, with supraphysiological doses of morphogens not being a viable alleviation to the problem.

## TGF-β superfamily members and bone formation

The work of Wozney et al. (1988) permitted the molecular cloning of several BMPs/OPs, which were found to be members of the TGF-β supergene family. Proteins were firstly isolated after chromatographic purification methods (Urist et al., 1984; Sampath et al., 1987; Wang et al., 1988; Luyten et al., 1989). Amino acid sequence motifs from batches of purified proteins were then used for molecular cloning of an entirely new family of proteins initiators, collectively called the BMPs (Wozney et al., 1988; Celeste et al., 1990; Özkaynak et al., 1990; Hammonds et al., 1991; Sampath et al., 1992). We have since then learned that BMPs are a family of highly conserved secreted pleitropic proteins that intiate cartilage and bone formation *in vivo* (Wozney et al., 1988; Sampath et al., 1992; Reddi, 2000; Ripamonti et al., 2004; Ripamonti, 2006).

BMPs have been shown to be involved in pattern formation during embryonic organogenesis (Reddi, 2005). A remarkable characteristic of BMPs gene products is that when implanted heterotopically in animal models they can induce new endochondral bone formation (Reddi, 2000; Ripamonti et al., 2004; Ripamonti, 2006).

## Bone morphogenetic proteins

Development, embryogenesis, pattern formation and organo-and skeletogenesis require the action of BMPs (Alliston and Derynck, 2000; Alliston et al., 2008). BMP signaling is regulated via their localized expression, the action of specific antagonists and a series of negative and positive feedback loops (Alliston and Derynck, 2000; Alliston et al., 2008). During skeletal development BMP-2 to -7 have an overlapping pattern of expression to ensure development occurs in an ordered manner (Solloway et al., 1998). Whilst BMP receptors type II and type I alpha (BMPRII and BMPRIA), like most members of the TGF-β family, are ubiquitously expressed (Kawabata et al., 1995), BMPR type I beta (BMPRIB), as shown by Ishidou et al. (1995), is principally transcribed only within developing bone and cartilage. Only when bone, in adult life, is damaged are all BMP receptors expressed (Onishi et al., 1998), as they facilitate part of the activation of the bone regeneration cycle. Subsequently, further research studies by Rickard et al. (1998), Mundy et al. (1999) and Rawadi et al. (2003) has shown that additional proteins, specifically statins, estrogen and wingless-type MMTV integration site family member 3A (Wnt3a), are also capable of activating the BMPs.

The activities of the BMPs are highly regulated by a series of antagonists and agonists. Chordin, Follistatin, Gremlin, Noggin and Sclerostin are specific antagonists known to inhibit the BMP pathway activation and thus bone formation (Alliston and Derynck, 2000; Alliston et al., 2008). Research studies have revealed BMPs are also capable of activating the expression of their inhibitors including Noggin and the intracellular BMP pathway inhibitory Smad family members 6 and 7 (Smad6 and Smad7) (Gazzerro et al., 1998; Takase et al., 1998; Ishisaki et al., 1999; Pereira et al., 2000; van Bezooijen et al., 2005). Additionally, members of the entire BMP signaling pathway, including their initiators and antagonist are subjected to further regulation by a series of positive and negative feedback signaling loops that interact with other signaling pathways (Alliston and Derynck, 2000; Alliston et al., 2008).

BMP signaling commences when a BMP ligand binds first to either the BMPR1A or BMPRIB, after which it forms a receptor complex with BMPRII, i.e., BMPRIA/B + BMPRII (Berk et al., 1997; Hayashi et al., 1997; Imamura et al., 1997; Yoshida et al., 2000; Sowa et al., 2004). This active complex then activates the Smad transcription receptor complex to regulate nuclear transcription factor activity (Berk et al., 1997; Hayashi et al., 1997; Imamura et al., 1997; Yoshida et al., 2000; Sowa et al., 2004). It is this spatial and temporal regulation of the various components of the BMP pathway that ensure proper skeleto-and organogenesis during embryo development as well as the maintenance of bone, later in postnatal life (Alliston and Derynck, 2000; Alliston et al., 2008).

Storm et al. (1994) revealed that growth differentiation factor (GDF)-5, -6, and -7, also referred to as cartilage-derived morphogenetic proteins (CDMP-1, -2, and -3) (Chang et al., 1994; Storm et al., 1994) are involved in bone formation and development *in vivo* (Mikic et al., 2002). GDF-5 to -7 have been found, via gene knocked studies, to control the length of long bone formation in limbs and regulate patterning of segments in the digits, chondrogenesis and longitudinal bone growth (Storm et al., 1994; Storm and Kingsley, 1996, 1999). GDFs have also been shown to induce osteogenic differentiation within *in vitro* stem cell differentiation studies (Erlacher et al., 1998; Yeh et al., 2005).

In term of bone formation by induction, the first of the BMPs to be used in induction studies was the recombinant human BMP-2 (rhBMP-2). In a time study, conducted by Wang et al. (1990), rhBMP-2 increased the rate of cellular invasion and induced chondrogenesis within the demineralized bone matrices after just 5 days. Within 7 days the cartilage was beginning to be ossified and after just 21 days the bone matrix had been ossified. These findings thus established that BMP-2 was one of the signals essential at initiating bone formation, be it within biomaterials or native bone regeneration respectively. Subsequently other BMPs were tested for their role in inducing bone formation. BMP-4 induces bone formation but only when used at high concentration (Hammonds et al., 1991), with BMP-5 inducing bone at a reduced rate, irrelevant of application dose (Cox et al., 1991; D'Alessandro et al., 1991). Subsequently BMP-6, osteogenic protein-1 (OP-1/BMP-7) and BMP-9 all are known to induce bone formation similar to that of BMP-2 (Riley et al., 1996). With such a substantial background of BMPs being an osteoinductive factor both in animal models and within subsequent clinical trials (Boden et al., 2002) it was postulated that bone formation was initiated and modulated only by the BMPs and their signaling pathway, since osteoinductive experiments before 1997 testing TGF-β isoforms, in rodents and lagomorphs, failed to induce bone formation (Roberts et al., 1986).

## Transforming growth factor-β isoforms

The three mammalian TGF-β isoforms, TGF-β_1_, TGF-β_2_, and TGF-β_3_ signal through receptor complexes of the TGF-β type II receptor (TßRII) and TßRI (Alliston et al., 2008). *In vitro* research has shown that although each of the TGF-β isoforms functions similarly at the cellular level (Alliston et al., 2008) their contribution within embryonic osteogenesis is quite different (Alliston et al., 2008). In knockout studies, during murine development, each corresponding gene produces unique phenotypic deviations, which reflect the TGF-β isoforms different spatio-temporal distributions and roles. The silencing of TGF-β_1_ results in abnormal bone quality (Shull et al., 1992; Kulkarni et al., 1993), whereas deletions of TGF-β_2_ or TGF-β_3_ impaires epithelial-mesenchymal trans-differentiation, cyto-proliferation and palate formation of the skull (Kaartinen et al., 1995; Proetzel et al., 1995; Sandford et al., 1997).

During bone formation in skeletogenesis, the expression patterns from various TGF-β isoforms are further modulated by a series of positive and negative feedback loops (Alliston et al., 2008), including the various inhibitors. However, there are apparent differences between the mRNA and protein expression patterns, which regulate TGF-β isoform storage in cartilage and bone (Alliston and Derynck, 2000). Pelton et al. (1991) indicated that the extracellular matrix proteins from bone interact with certain of TGF-β isoforms, particularly TGF-β_1_, allowing them to be deposited within bone. In the mesenchyme, all TGF-β isoforms are expressed which is increased as the mesenchyme condenses in preparation for osteogenesis (Alliston et al., 2008). Experimental research has revealed that whilst TGF-β_3_ is expressed during the early development of cartilage formation, TGF-β_1_ and TGF-β_2_ expression is hardly transcribed (Pelton et al., 1990, 1991). As osteogenesis matures, TGF-β_3_ levels diminish, whereas TGF-β_1_ and TGF-β_2_ levels increase (Pelton et al., 1990). Primarily it seems that TGF-β_3_ levels are highest in tissues associated with skeletogenesis and bone formation, whereas TGF-β_2_ more likely to be transcribed at sites of where new bone mineralization occurs (Alliston et al., 2008).

In previous research studies of mouse cartilage, TGF-β_3_ expression is higher than in relation to the other TGF-β isoforms (Pelton et al., 1990, 1991). Subsequently other research studies on the mouse growth plate have found that whilst TGF-β_2_ is transcribed throughout all the regions of the developing plate, TGF-β_1_ and TGF-β_3_ are restricted to only the hypertrophic and proliferative areas (Sandberg et al., 1988; Millan et al., 1991; Thorp et al., 1992; Horner et al., 1998). The periosteums of endochondral and intra-membranous bones shows high levels of expression of TGF-β_1_ and TGF-β_2_ respectively, whilst osteoclasts and osteoblast show varied expression patterns in TGF-β isoforms (Pelton et al., 1991).

In developing bones the TβRI and TβRII are transcribed throughout the developing embryonic skeleton (Horner et al., 1998), but in hypertrophic chondrocytes and mineralized osteophyte tissue their expression is reduced and lost, respectively (Horner et al., 1998). The Smads, known regulators of the TGF-β pathway, are also subject to regulation in skeletal tissue (Sakou et al., 1999). *In vitro* cell culture experiments Smad2, Smad3, and Smad4 including the inhibitory Smad6 and 7 are all differentially expressed within proliferating chondrocytes and bone cells (Sakou et al., 1999; Alliston et al., 2008).

Inactivation and storage of TGF-β isoforms is facilitated by latent TGF-β-binding proteins (LTBPs) (Shipley et al., 2000), where all four isoforms of LTBPs-(1-4), except for LTBP-2 associate with the TGF-β isoforms (Shipley et al., 2000). Although all four LTBPs are broadly expressed and regulated, LPBT-3 expression is the highest in bone, whereas LTBP-2 has been detected to be principally expressed in chondrogenic condensations (Shipley et al., 2000). Although other studies have shown that there are ways in which the formation and storage of TGF-β complexes can be regulated (Oreffo et al., 1989; Oursler, 1994; Dallas et al., 2002; Kwok et al., 2005), bone formation by the LTBPs isoforms is considered to be the primary mode by which skeletal bone is developed.

With respect to bone formation by induction, TGF-β isoforms were shown in well-established animal models, such as the rat, to be non-osteoinductive (Roberts et al., 1986), instead tending to form endothelial or vascular tissue. However, when TGF-β_1_ was loaded within a collagen-matrix carrier and implanted within heterotopic extraskeletal muscle sites of the rectus abdominis muscle, of *Papio ursinus* (Chacma baboon), bone formation was induced (Ripamonti et al., 1997). In a series of systematic studies, in this new animal model, Ripamonti et al. (2000, 2008) showed that the principle concept of BMP induced bone formation to be flawed, by showing that also TGF-β_2_ and especially TGF-β_3_ could induce bone formation with biomimetic devices in these animals.

Naturally this theory has since then been further adapted, as more recent studies in non-human primates and monitoring the gene expression over a period of 90 days has revealed that the supposed induction of bone formation by TGF-β_3_ in fact is not direct but regulatory. The TGF-β_3_ isoform seems to act as a major regulator, a suggested signaling control center, which regulates BMP signaling by down regulating inhibitory BMP signals (Klar et al., 2014). Thus rather than directly affecting the induction process within a heterotopic implantation model, the TGF-β_3_ isoform modulates specialized molecular and cellular cues and therefore can indirectly initiate the induction process by allowing the over-expression of BMPs to occur, resulting in not just increased induction rates but also quicker formation of bone through heightened cellular differentiation and proliferation (Klar et al., 2014). Subsequently, and more interesting, is the fact that the signals of the TGF-β isoform family evolved or mutated somewhere, between rats and primates as the effect of TGF-β isoforms seems to behave differently between these two species (Ripamonti et al., 2016). This suggests that homogeneity principles applied between animal models and humans must also be inconsistent, which are indeed supported by the in-translatability of bone studies from animals into humans. Could this be part of the problem and solution or does this mean that indeed all proteins, whilst inherently similar in structure function differently the higher one goes in evolution? The issue remains that of clinical translatability, where neither BMPs/OPs nor TGF-β isoforms have shown any significant impact similar or better than the autogenous bone graft.

## The animal translation enigma

Evidence has always indicated that *in vitro* to *in vivo* testing and subsequently *in vivo* to human trials do not properly replicate treatments within a clinical setting (Denayer et al., 2014). This suggests that key factors are not considered in attempts to translate animal experimentation principles into a clinical setting.

Major areas of concern are those of pathophysiology, methodological design and sample size variations which are often ignored when translating into the clinical aspect, whilst others are limited to the extent of ethical concerns when experimenting on humans. In their review Denayer et al. (2014) adequately list critical determinants when it comes to translating to a human model. Evans (2016) also highlights criteria such as maturity of animals, scale-up from animal models to humans and differences between bones, i.e., cranial vs. femur. Alternatively, it has to be considered that present bone induction procedures are utilizing allo-and xenografting principles, that have been shown to not function *in vivo* (Ladd and Pliam, 1999; Keating and McQueen, 2001; Betz, 2002; Linovitz and Peppers, 2002), but are replicated in the form of biomaterials, utilizing autogenous bone grafting principles.

Another critical mistake is that most studies overlook the difference between animal and human genes that, whilst structurally similar, have different expression patterns or function. Often it is assumed that animal models, in particular for inductive bone studies, are genetically compatible to each other since osteogenesis appears to be similar between all experimentally utilized animal models compared to humans. Whilst there are homologous trends in the gene structure of various animal models, with that of the human including functionality *in vivo*, a fact often left out is that in most cases subtle variations in gene structure can produce considerable difference between species. The research of Ripamonti et al. (1997, 2000, 2008, 2016) and TGF-β isoforms only inducing bone formation in non-human primates opposed to other animal models (Roberts et al., 1986), clearly reiterates this. However the main causes for these conflicting results are perhaps related more to the trend of not presenting unexpected results, together with the use of outdated methods. A good example here would be quantitative real-time reverse transcription polymerase chain reaction (qRT-PCR).

qRT-PCR has become the leading analytic technique which over the last two decade has made considerable progress (Mullis, 1990; Higuchi et al., 1993; Bustin, 2000, 2002; Vandesompele et al., 2002; Bustin and Nolan, 2004; Bustin et al., 2009, 2010, 2013; Vermeulen et al., 2011) at improving the accuracy of gene expression data. Whilst some bone and cartilage research groups have already utilized the new standards as set out by Bustin et al. (2009) pioneering “Minimum Information for Publication of Quantitative Real-Time PCR Experiments (MIQE) guidelines” (Bustin et al., 2009; Klar et al., 2013, 2014; Ripamonti et al., 2016), a considerable number of research groups in the bone tissue engineering field still utilize outdated and highly insufficient qRT-PCR techniques that, when combined with frequent insufficient experimental detail, render replication of many published findings challenging (Sanders et al., 2014) and very questionable.

## Autogenous biomaterial composition enigma

Though much research has been conducted into understanding how to induce new bone formation and which growth factors show distinctive patterns to initiate, promote, regulate and modulate new bone formation, single dosages of these morphogens simply do not produce the necessary amount of bone formation required to make these procedures standard clinical practice.

To this day few studies have attempted to deviate from this practice. To date Ripamonti et al. (1997, 2010) has been the only study on bone formation by induction to show that two separate but structurally similar TGF-β supergene family members could synergize with each other to increase bone formation quantities *in vivo* compared to utilizing conventional single morphogen applications of the same type. However, more needs to be done. Taken in consideration with an autogenous bone graft the following remains elusive.

First the autogenous bone graft contains a reservoir of structural and signaling proteins; the second is that these proteins are present at a physiological dose and in a “non-recombinant” state. A possible reason why an autogenous bone graft works is that it possesses complete and specific protein content. From these are initiators, regulators, inhibitors, modulators including all the structural molecules, arranged in a certain configuration in the 3D superstructure of the graft which together interacts with each other and subsequently with site to where the graft is transplanted. The healing equilibrium is therefore maintained, compared to being greatly limited if only a single morphogen were present. Indeed, Wildemann et al. (2007) described this in part, but the findings were restricted to only assessing the content of proteins that were present at the highest concentrations. The complete protein content of a fragment of bone from different skeletal areas remains elusive.

Another determining factor, aside from the types of proteins, is the exact protein quantity of each protein within a fragment of bone. At the incorrect dosage, BMPs and TGF-βs in the bone induction have been shown to stimulate the formation of tumors and cancers (Riemenschneider et al., 2015; Skovrlj et al., 2015). Zara et al. (2011) has shown that the correct amounts of protein-induced bone formation exist at significantly lower levels than is currently utilized to induce new formation of bone within *in vivo* and clinical models. Subsequently, all forms of proteins utilized in the scientific bone tissue engineering field are recombinant proteins, which are not the same structure as found natively within the bone matrix. Recombinant human BMP-2, for example, is a dimeric protein whereas in its native form is a heterodimer (Israel et al., 1996; Evans, 2016). It is suggested that the heterodimer functions differently at inducing bone formation than the recombinant molecule where the heterodimeric structure resists total inhibition by modulators, such as Noggin (Song et al., 2010) to permit for more regulated bone formation. The autograft as such could be working because the amounts and correct protein structures are available in a configuration suitable for the natural physiological program to decode, supporting the physiological response, thereby allowing for proper re-formation of bone at the defect site.

## Future research recommendations

Whilst many relevant concepts and theories exist, their implementation into a clinical scenario is based on how accurate results are, irrelevant of the type of synthetic induction model considered. When Urist, Reddi, Sampath and Wozney (Urist, 1965; Sampath and Reddi, 1981; Wozney et al., 1988) originally started deciphering the induction of bone formation, the technology of molecular biology was very limited. Whilst much has been done in the field of bone tissue engineering, it is becoming clear that fundamental core foundations need to be re-assessed (Evans, 2016) and updated.

In order to reverse engineer a complex system, one must take it apart into all of its components; assess the shape and function of these constituents, how they integrate within each other and how each component affects their neighbors. Following this, cloned components can be engineered synthetically with most of the original traits and reassembled to resemble the original system in shape and function (Chikofsky and Gross, 1990; Eldad, 2005). Overlooking one component's design feature will lead to misbehaving replica, meaning a revision of theories and paradigms until parts are adequately designed and work as they are intended to. Taken into the context of the bone formation by induction vs. autogenous bone grafting, the problem remains evidently complex and multi-faceted, where there are many solutions possible too numerous to contain all within a single review.

Critical is that future research needs to accept that as technology improves, old paradigms need to be updated based on previous mistakes. Specifically toward solving bone formation by induction, in the opinion of the author, a detailed library of all micro-and macromolecules, which include also other molecules such as miRNAs, nano-and macro-particles, including how these are arranged within the superstructure of all skeletal bones, needs to be established to permit accurate synthetic replication of the healing processes within *in vivo* clinical applications. Subsequently, while current animal models have shown us some insights, they are unreliable and are unsuitable for developing treatments for bone tissue engineering. There is a clear need to develop more reliable non-*in vivo* models that adequately replicate the human bone environment *in vitro* allowing for faster and more accurate clinically transducable treatments to be developed. Here, bioreactors platforms, replicating bone as an organ, are the perfect alternative to simulate the *in vivo* human environment (Martin et al., 2004; Plunkett and O'Brien, 2011), provided however that the library of all micro- and macromolecules exists. Only by critically re-evaluating the past in light of the future can bone formation without autogenous bone grafting become feasible clinically.

## Author contributions

The author confirms being the sole contributor of this work and approved it for publication.

### Conflict of interest statement

The author declares that the research was conducted in the absence of any commercial or financial relationships that could be construed as a potential conflict of interest. The reviewer AS and handling Editor declared their shared affiliation
